# Author Correction: Photonic Spin Hall Effect in Waveguides Composed of Two Types of Single-Negative Metamaterials

**DOI:** 10.1038/s41598-019-42460-y

**Published:** 2019-04-17

**Authors:** Zhiwei Guo, Haitao Jiang, Yang Long, Kun Yu, Jie Ren, Chunhua Xue, Hong Chen

**Affiliations:** 1 Key Laboratory of Advanced Micro-structure Materials, MOE, School of Physics Science and Engineering, Tongji University, Shanghai, 200092 China; 2 Center for Phononics and Thermal Energy Science, School of Physics Science and Engineering, Tongji University, Shanghai, 200092 China; 3 School of Computer Science & Communication Engineering, Guangxi University of Science and Technology, Liuzhou, Guangxi 545006 China

Correction to: *Scientific Reports* 10.1038/s41598-017-08171-y, published online 10 August 2017

In the original version of this Article, Affiliation 2 was incorrectly listed as ‘Institute for Advanced Study, Tongji University, Shanghai, 200092, China’. The correct affiliation is listed below:

Center for Phononics and Thermal Energy Science, School of Physics Science and Engineering, Tongji University, Shanghai, 200092, China

In addition, Yang Long was incorrectly affiliated with ‘Key Laboratory of Advanced Micro-structure Materials, MOE, School of Physics Science and Engineering, Tongji University, Shanghai, 200092, China’. The correct affiliation is listed below:

Center for Phononics and Thermal Energy Science, School of Physics Science and Engineering, Tongji University, Shanghai, 200092, China

These errors have now been corrected in the PDF and HTML versions of the Article.

